# Mining glycosylation-related prognostic lncRNAs and constructing a prognostic model for overall survival prediction in glioma: A study based on bioinformatics analysis

**DOI:** 10.1097/MD.0000000000033569

**Published:** 2023-05-05

**Authors:** Xiang Wu, Haiyan Wang, Shiqi Li, Haitao Luo, Feng Liu

**Affiliations:** a Department of Neurosurgery, Jiangxi Provincial Children’s Hospital, Nanchang, China; b Institute of Neuroscience, Nanchang University, Nanchang, China; c Department of Operation, The Second Affiliated Hospital of Nanchang University; d Department of Neurosurgery, The Second Affiliated Hospital of Nanchang University, Nanchang, Jiangxi Province, China.

**Keywords:** CGGA, glioma, glycosylation, lncRNA, TCGA

## Abstract

Dysregulation of protein glycosylation plays a crucial role in the development of glioma. Long noncoding RNA (lncRNAs), functional RNA molecules without protein-coding ability, regulate gene expression and participate in malignant glioma progression. However, it remains unclear how lncRNAs are involved in glycosylation glioma malignancy. Identification of prognostic glycosylation-related lncRNAs in gliomas is necessary. We collected RNA-seq data and clinicopathological information of glioma patients from the cancer genome atlas and Chinese glioma genome atlas. We used the “limma” package to explore glycosylation-related gene and screened related lncRNAs from abnormally glycosylated genes. Using univariate Cox analyses Regression and least absolute shrinkage and selection operator analyses, we constructed a risk signature with 7 glycosylation-related lncRNAs. Based on the median risk score (RS), patients with gliomas were divided into low- and high-risk subgroups with different overall survival rates. Univariate and multivariate Cox analyses regression analyses were performed to assess the independent prognostic ability of the RS. Twenty glycosylation-related lncRNAs were identified by univariate Cox regression analyses. Two glioma subgroups were identified using consistent protein clustering, with the prognosis of the former being better than that of the latter. Least absolute shrinkage and selection operator analysis identified 7 survival RSs for glycosylation-related lncRNAs, which were identified as independent prognostic markers and predictors of glioma clinicopathological features. Glycosylation-related lncRNAs play an important role in the malignant development of gliomas and may help guide treatment options.

## 1. Introduction

Among malignant tumors of the central nervous system, glioblastoma (GBM) is known for its high mortality rate, high recurrence rate, high malignancy degree, and poor prognosis.^[1,2]^ GBM accounts for 70% to 75% of all diagnoses of diffuse glioma,^[3,4]^ and its median overall survival (OS) time is 14 to 17 months,^[5,6]^ the 5-year survival rate is <5%.^[7]^ Conventional surgical treatment, postoperative radiotherapy and chemotherapy for GBM has limitations.^[8]^ Therefore, GBM is considered to be one of the most challenging malignant tumors worldwide. The prime cause of glioma’s invasiveness is credited to the abnormal amounts of glycoproteins and glycolipids, as well as the effect of aberrant glycosylation on the conformation, concentration of adhesion molecules, and the proteolytic enzymes in the microenvironment of the tumor. In addition, the extracellular matrix (ECM) is also affected.^[9]^ Abnormal glycosylation is associated with the underlying mechanism of tumorigenesis and metastasis.^[10]^ The metastasis and invasion of gliomas regulate intercellular interactions by altering the ECM.^[11]^ Moreover, structural changes on the surface of glycosylated cells cause glioma cells or adjacent cells to abnormally secrete cell surface and secretory proteins such as ECM glycoprotein cytokines and growth factors. Glycosylation alterations promote interactions between tumor cells and contact dependence, allowing tumor cells to invade distal tissues.^[12,13]^

Protein glycosylation is the covalent attachment of 1 or more glycogens and biomolecules to a nascent polypeptide chain through enzymes.^[14]^ This process can connect monosaccharides or complex polysaccharides to the protein side chains. It affects more than half of the known proteins by altering interactions between them or via indirect mechanisms such as protein conformation, stability, and conversion rate, thereby affecting the invasion, proliferation, immunity, and radiation sensitivity of gliomas.^[12,15]^ For example, N-acetylgalactosamine transferase 2, an enzyme that regulates the initial steps of mucin O-glycation, promotes the proliferation and growth of gliomas by affecting the O-glycation and phosphorylation of EGFR and its subsequent downstream PI3K/Akt/mTOR axis.^[16]^ The expression of MUC4 (a highly O-glycated protein) may be involved in the proliferation and invasion of GBM cells by upregulating EGFR.^[17]^ Glycosylated programmed death ligand 1 and its homologous receptor PD1 are involved in the inhibition of T cell activation by kinase inhibition mediated by phosphatase SHP2 in glioma, thus protecting the tumor from the influence of the immune microenvironment.^[18]^ Inhibition of oligosaccharide transferase (NGI-1) can reduce the activation of receptor tyrosine kinases and enhance glioma radiosensitivity.^[19]^ The occurrence, development and metastasis of gliomas are closely related to protein glycosylation.^[20]^

Long noncoding RNAs (lncRNAs) are more than 200 bp in length and have no protein-coding function.^[21,22]^ However, an increasing number of studies have found that lncRNAs play carcinogenic or inhibitory role in the development of malignant tumors.^[23]^ Several lncRNAs are abnormally expressed in gliomas. For example, the transcription factor NBAT-1NRSF/REST, which activates neuronal specificity, promotes the invasiveness of nerve GBM.^[24]^ Foxm1-as promotes the interaction between ALKBH5 and FOXM1 mRNA and the demethylation of FOXM1 mRNA and enhances the self-renewal and tumorigenesis of GBM cells.^[25]^

An increasing number of studies have shown that changes in glycoproteins levels are indicative of cancer. Therefore, it is of great significance to study the molecular mechanisms of glioma and identify targets related to glycosylation for the treatment of glioma. In this study, we screened glycosylation-related lncRNAs in gliomas and explored the relationship between the glycosylation lncRNAs and prognosis of glioma patients.

## 2. Materials and Methods

### 2.1. Data collection

Human glioma (GBM, low-grade glioma [LGG]) RNA-seq data and clinical information were collected from the Chinese glioma genome atlas (CGGA) (http://www.cgga.org.cn/) (n = 553) and the cancer genome atlas (TCGA) (http://cancergenome.nih.gov/) (n = 546).

### 2.2. Screening glycosylation-related lncRNAs

LncRNAs from TCGA and CGGA databases were screened via the Ensembl database (https://www.ensembl.org/) and glycosylation-related genes were screened using gene set enrichment analysis. The overlapping glycosylation-related genes were screened based on TCGA and CGGA datasets, and abnormally glycosylated genes with a *P* < .05 were analyzed to determine different expressions between GBM and LGG. The Pearson correlation analysis was used to identify lncRNAs related to abnormal glycosylation and whose *P* < .001 and correlation coefficient was >0.5 or less than −0.5 in the 2 datasets. We performed univariate Cox regression analysis and screened 20 overlapping lncRNAs in both the CGGA and TCGA datasets for subsequent analysis.

### 2.3. Co-expression analysis of glycosylation-related lncRNAs

To determine the strength of the relationships between the transcriptional levels of these lncRNAs, we determined the co-expression relationship between these lncRNAs related to abnormal glycosylation according to the RNA-seq data and used the “corrplot” package in R software to calculate the Pearson correlation coefficient of these lncRNAs.

### 2.4. Bioinformatics analysis

To explore the functions of the glycosylation-related lncRNAs, we divided gliomas into various clusters by using the “Consensus Cluster Plus” package (50 iterations, 80% resampling rate, Pearson correlation, http://www.bioconductor.org/). Principal component analysis (PCA) was carried out with “R” to obtain the expression patterns of significant glycosylation-related lncRNAs in various clusters.

### 2.5. Construction of the least absolute shrinkage and selection operator (LASSO) regression model and verification of survival risk

The 20 over lapping glycosylation-related lncRNAs from TCGA dataset were incorporated into the LASSO regression analysis to identify prognostic biomarkers.^[26–28]^ We used the LASSO analysis to determine the weight of selected lncRNA coefficients. A risk score (RS) formula was constructed as follows: $RS=\sum_{i=1}^{n}\left(Coef\;i*xi\right)$.

The median RS was used to divide the lncRNAs into high and low-risk groups. PCA was carried out with “R” to obtain the expression patterns of the significant glycosylation-related lncRNAs in the low-risk and high-risk groups. Kaplan–Meier (KM) curves were generated to determine the survival statuses of glioma patients in the low- and high-risk groups. The “timeROC” package was used to draw the receiver operating characteristic (ROC) curves of each dataset separately.

### 2.6. Validation of the RS model

To validate the validity and reliability of our glycosylation-related lncRNA RS formula, KM and time-dependent ROC curve analyses were conducted on the CGGA validation dataset.

### 2.7. Expression and survival analysis of the glycosylation-related lncRNAs

For the 7 glycosylation-related lncRNAs screened via LASSO regression analysis, we constructed a violin diagram to visualize the trend in gene expression levels and compared their mRNA expression levels at different stages based on TCGA database. We also analyzed the associations of the 7 lncRNAs in the TCGA dataset with survival and verified their associations with the CGGA dataset.

### 2.8. Patients and tissues

From October 2019 to June 2020, glioma tissues were collected from 24 glioma patients (pathological types WHO II, WHO III and WHO IV, 8 patients for each type) who underwent surgery at the Second Affiliated Hospital of Nanchang University, and normal brain tissues were collected from 6 normal patients who underwent cerebral hemorrhage surgery. All specimens were frozen in liquid nitrogen immediately after surgery and stored at −80°C. The research protocol was approved by the Second Affiliated Hospital of Nanchang University, and adhered to the ethical guidelines of the 1975 Declaration of Helsinki. The use of gliomas and normal brain tissues were approved by the institutional ethics committee of Nanchang University. All patients enrolled in this study provided written informed consent.

### 2.9. RNA extraction and qRT-PCR

TRIzol (Invitrogen) was used to extract lncRNA and total RNA from glioma and normal brain tissues according to the manufacturer’s protocol. DNase I (Roche, Indianapolis, IN) was used to remove residual DNA. qRT-PCR was performed to detect the expression of AC083799.1, AC062021.1, SNHG6, LINC00507, FAM66C, DGCR10, and LINC00641. A cDNA synthesis kit (TaKaRa Biotechnology Co., Ltd., Dalian, China) and oligo (dT) primers were applied to reverse transcribe RNA (2 μg) to cDNA, and SYBR Green qPCR Master Mix (TaKaRa) was used for qRT-PCR according to the manufacturer’s instructions. GAPDH was used for normalization. The oligonucleotides used as PCR primers are shown in Table S1, Supplemental Digital Content, http://links.lww.com/MD/I819, PCR was performed on the ABI 7300 system (Applied Biosystems, Foster City, CA) as follows: initial incubation at 95°C for 10 minutes, followed by 95°C for 10 minutes and 40 cycles at 95°C for 15s and 60°C for 45s. The 2−ΔΔ Ct method was employed to calculate the fold changes. All data represent the average of 3 replicates. The results were used to verify the expression levels of the 7 lncRNAs related to abnormal glycosylation in tumors of different grades.

### 2.10. Statistical analysis

The expression of glycosylation-related genes and their relationship with GBM and LGG were analyzed using analysis of variance. The *t* test was used to compare the age, sex, IDH status, and 1p/19q codel status of the glioma patients. The patients were divided into low-and high-risk groups using the median RS as the cutoff value. The chi-squared test was used to compare the clinical and molecular pathological distributions of the 2 risk groups. We conducted 1-way analyzed using analysis of variance or t-test to compare RSs of patients grouped by clinical or molecular pathological characteristics. The ROC curve was used to determine the prediction efficiency of the RS, WHO classification, survival age, G1/2 group, and IDH mutation status, and 1p/19q codel status. Univariate and multivariate Cox regression analyses were performed to confirm the prognostic value of RS and various clinicopathological features, including RS, age, sex, IDH status, and 1p/19q codel status. The KM method of the bilateral log-rank test was used to investigate the OS outcomes of patients in the G1/2 and the high and low-risk groups. qRT-PCR was performed using the 2−ΔΔ Ct method to calculate fold change. All statistical analyses were performed by using R v3.6.1 (https://www.r-project.org/), SPSS 16.0 (SPSS Inc., Chicago, IL), and Prism 8 (GraphPad Software Inc., La Jolla, CA). The statistical significance of all data is indicated in the figures as follows: *P < .05, **P < .01, ***P < .001, and ****P < .0001.

## 3. Results

### 3.1. Obtaining glycosylation-related lncRNAs

**Figure 1.**
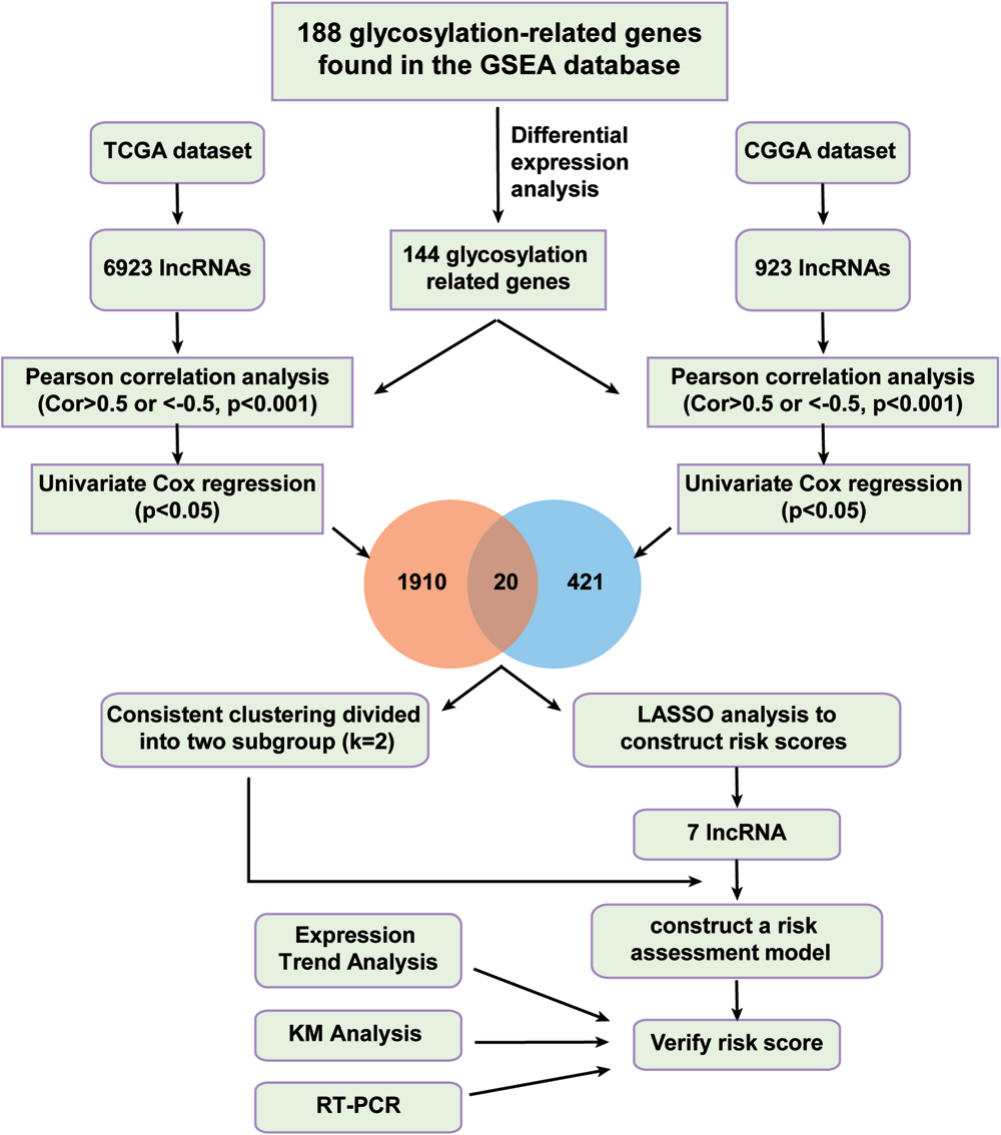
The whole process of this study. The abnormal glycosylated gene-associated lncRNAs were screened out based on TCGA and CGGA datasets, then we employed LASSO analysis to construct a risk profile model incorporating clinicopathological information and ascertain its independent prognostic power. CGGA = Chinese glioma genome atlas, LASSO = least absolute shrinkage and selection operator, lncRNAs = long noncoding RNA, TCGA = the cancer genome atlas.

**Figure 2.**
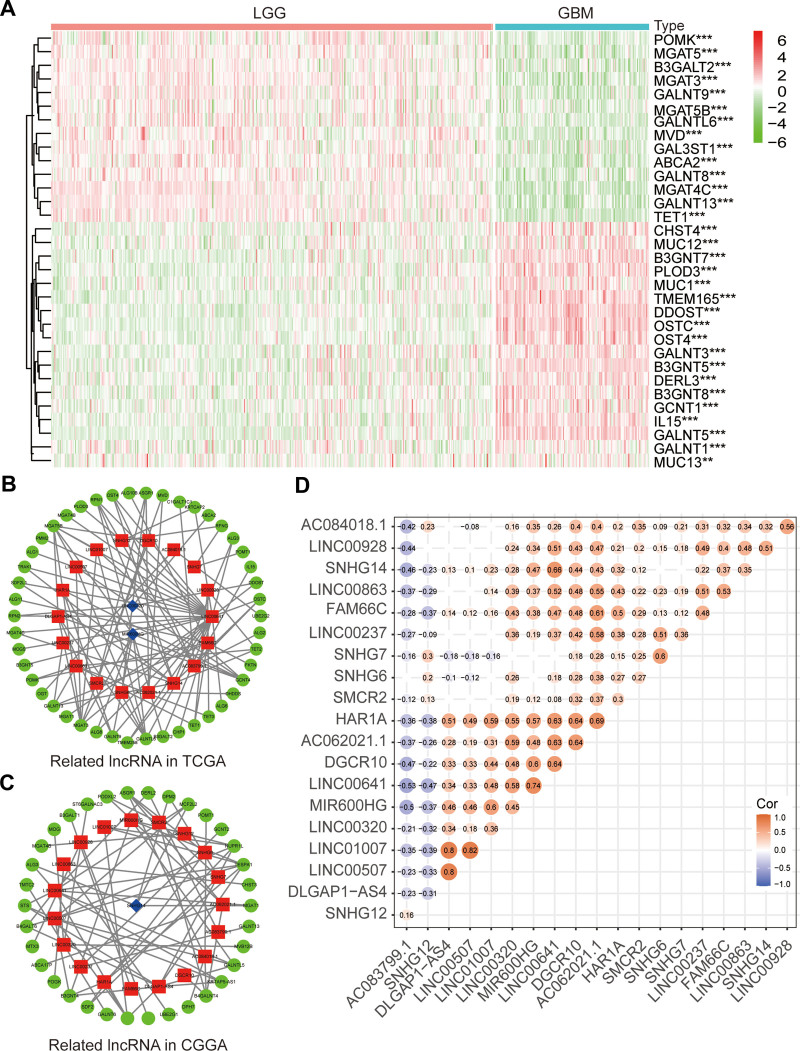
Mining glycosylation-related lncRNAs and their correlation analysis. (A) Expression of glycosylation-related genes in gliomas with different clinicopathological features. (B and C) The correlation network structure between glycosylation-related lncRNAs and genes in TCGA (B) and CGGA, (C) red represents upregulated and blue downregulated genes. (D) The correlations of the twenty lncRNAs. CGGA = Chinese glioma genome atlas, lncRNAs = long noncoding RNA, TCGA = the cancer genome atlas.

The study process is shown in Figure 1. We identified 188 glycosylation-related genes using gene set enrichment analysis (Table S2, Supplemental Digital Content, http://links.lww.com/MD/I820). We identified 144 abnormally glycosylated genes in LGG and GBM through differential analysis (Table S3, Supplemental Digital Content, http://links.lww.com/MD/I821). Our differential analysis showed that most of the glycosylation-related genes were significantly related to the WHO grade. Only 32 of the most differentially expressed genes are shown in the heat map, of which POMK, MGAT5, B3GALT2, MGAT3, GALNT9, MGAT5B, and GALNTL6 and other glycosylation-related genes were negatively correlated with the WHO grade. The expression levels of glycosylated-related genes such as CHST4, MUC12, B3GNT7, PLOD3, MUC1, and TMEM165 were positively correlated with WHO grade (Fig. 2A). Screening (via Pearson correlation analysis; *P* < .001, Cor > 0.5, or < -0.5) revealed 1910 lncRNAs related to abnormal glycation in TCGA database and 420 lncRNAs related to abnormal glycation in CGGA database. Moreover, lncRNAs related to survival and abnormal glycosylation were screened via the univariate analysis (*P* < .05). The univariate analysis results obtained from the TCGA and CGGA databases are shown in Table S4, Supplemental Digital Content, http://links.lww.com/MD/I822 and Table S5, Supplemental Digital Content, http://links.lww.com/MD/I823. We also selected 20 lncRNAs related to abnormal glycosylation including LINC00863, DLGAP1-AS4, FAM66C, SNHG6, and LINC00507, which were provided in TCGA and CGGA databases. The co-expression network of these 20 glycosylation-related lncRNAs from TCGA and CGGA is shown in Figure 2B and C. To understand the interactions between these 20 glycosylation-related lncRNAs more clearly, we also analyzed their correlations. The results showed a significant correlation between them (Fig. 2D), indicating that the lncRNAs associated with abnormal glycosylation could be clustered into different groups. For example, among the 20 lncRNAs associated with abnormal glycosylation, DLGAP1-AS4 was positively correlated with LINC00507 and LINC01007, and AC083799.1 was negatively correlated with MIR600HG and LINC00641. This suggests that they may act in concert by regulating the same glycosylation process; however, this requires further exploration.

### 3.2. Consistent clustering of lncRNAs related to abnormal glycation divides glioma specimens into two clusters with different pathological features and clinical outcomes

**Figure 3.**
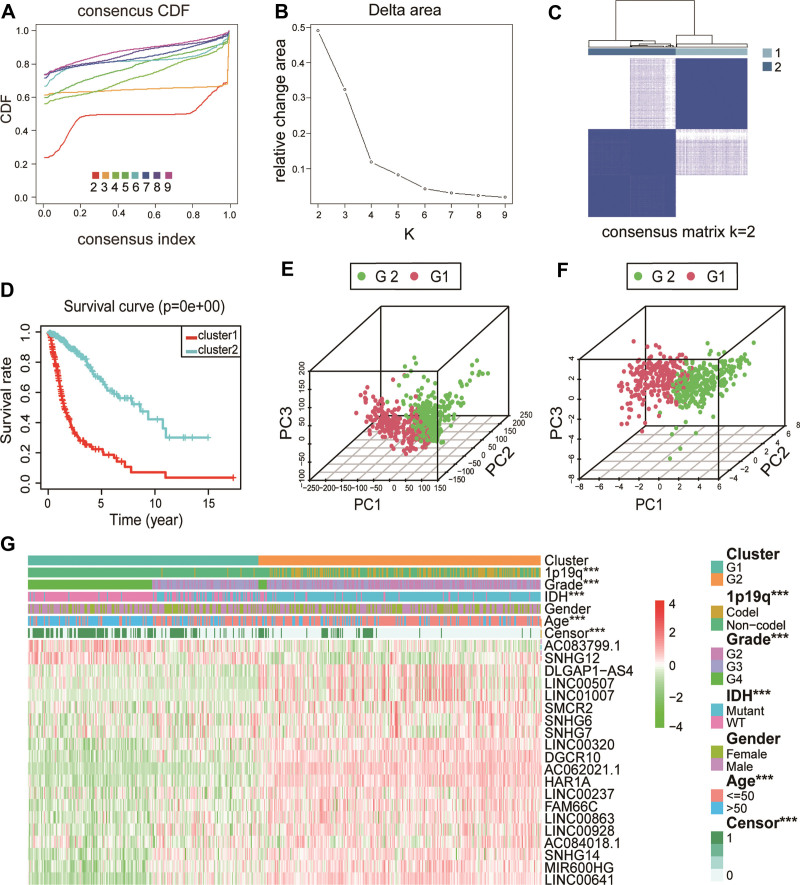
The distinction of clinicopathological features and OS of glioma patients in the G1/2 subgroups. (A) Consensus clustering cumulative distribution function (CDF) for k = 2–9. (B) Relative change in area under the CDF curve for k = 2–9. (C) Correlation between the G1 and G2 subgroups. (D) Kaplan–Meier overall survival (OS) curves for 547 TCGA samples. (E) Principal component analysis based on all genes of the dataset from TCGA. (F) Principal component analysis based on 20 glycosylation-related in TCGA. (G) Heatmap and clinicopathologic features of the 2 clusters (G1/2) defined by glycosylation-related lncRNAs consensus expression. lncRNAs = long noncoding RNA, TCGA = the cancer genome atlas.

The parameter “k = 9” was used to specify the maximum number of cluster groups expected to be divided. Therefore, beginning from cluster 2, we increased the number of categories 1 by 1 until we reached the specified maximum number of cluster groups of 9. To determine the most appropriate number of clusters, the inter-group correlation between the CDF values in Figure 3A and B and the groups in Figure 3C was evaluated; ultimately, a value of k = 2 was selected (Fig. 3A–C). We divided the 546 glioma samples from the TCGA dataset into 2 subgroups: G1 and G2 (Table S6, Supplemental Digital Content, http://links.lww.com/MD/I824). We further compared the clinicopathological characteristics of the 2 subgroups. The G2 subgroup was associated with young age (*P* < .001) and low tumor grade (*P* < .001) at diagnosis, while the G1 subgroup was associated with old age and high tumor grade at diagnosis (Fig. 3G). It is interesting that the OS time of the G1 subgroup was shorter than that of the G2 subgroup (Fig. 3D). PCA was performed to compare the transcription profiles of the G1 and G2 subgroups. The results showed significant differences between G1 and G2. Figure 3E shows the PCA results for all genes. Figure 3F shows the PCA results for the 20 lncRNAs related to abnormal glycosylation. The results of our study demonstrated that 20 lncRNAs, namely, AC083799.1, AC062021.1, LINC00507, DGCR10, and so forth, were significantly expressed between G1 and G2 patients (Fig. 3G). Notably, these lncRNAs were associated with WHO grade (*P* < .001), age (*P* < .001), IDH status (*P* < .001) and 1p/19q codelet status (*P* < .001). This implies that the combination of selected lncRNAs and G1/G2 models which we established are expected to facilitate accurate prognosis for glioma patients.

### 3.3. Construction of the RS signature via LASSO regression analysis

**Figure 4.**
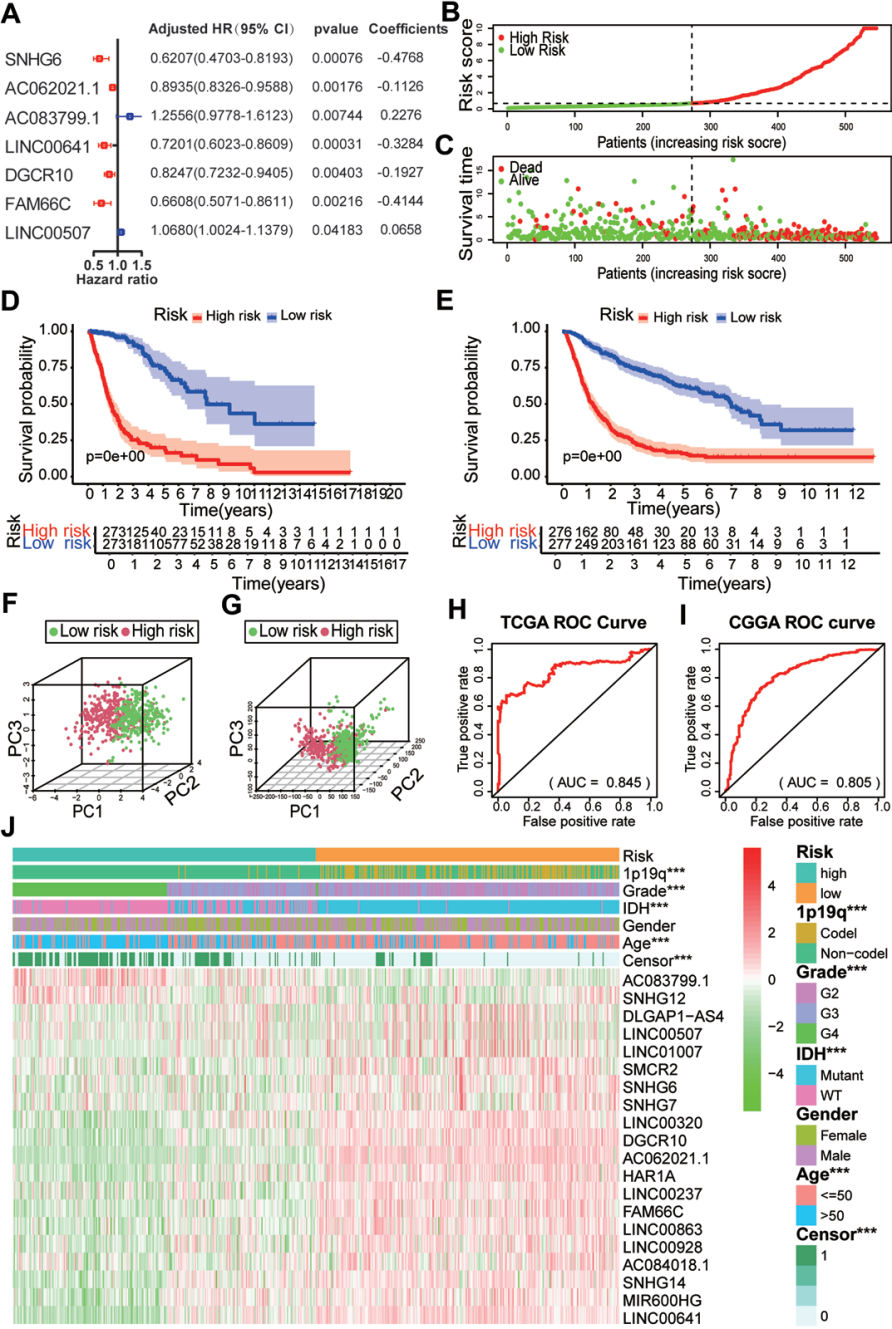
Risk signature with 7 glycosylation-related lncRNAs. (A) Seven survival-related glycosylation lncRNAs in the prognostic classifier associated with overall survival in TCGA. (B) Distribution of RS in TCGA. (C) Survival time and status of patients in TCGA. (D and E) Kaplan–Meier survival based on the 7 survival-related glycosylation lncRNAs in TCGA (D) and CGGA (E). (F) PCA plot based on all genes of the dataset from TCGA. (G) PCA plot based on 7 survival-related glycosylation lncRNAs in TCGA. (H and I) ROC curves of the risk signature in CGGA (H) and TCGA (I) datasets. (J). The heatmap of the twenty glycosylation-related lncRNAs in low- and high-risk gliomas. The distribution of clinicopathological features was compared between the low- and high-risk groups. *P < .05 and ***P < .001. CGGA = Chinese glioma genome atlas, lncRNAs = long noncoding RNA, PCA = Principal component analysis, ROC = receiver operating characteristic, RS = risk score, TCGA = the cancer genome atlas.

To verify the association between lncRNAs related to abnormal glycosylation and the survival of glioma patients, the 20 lncRNAs related to abnormal glycosylation were subjected to LASSO regression analysis. Seven of the 20 lncRNAs were significantly.

associated with OS (*P* < .05), as shown in Figure 4A. The samples were then divided into high-risk and low-risk groups according to the median RS (Fig. 4B). Glioma patients with a low RS had a higher survival rate than those with a high RS (Fig. 4C).

### 3.4. RS is an accurate indicator of prognosis

KM analysis also revealed that the survival rate of patients with a high RS was lower than that of patients with a low RS (*P* < .001, Fig. 4D). PCA was performed to compare the transcription profiles of patients with high and low RS. Figure 4F shows the PCA results for the 7 lncRNAs related to abnormal glycosylation.

Figure 4G shows the PCA results for all genes. The high and low-risk groups are represented by red and green dots, respectively. The results showed significant differences between the high and low-risk groups. The ROC curve suggested that RS could accurately predict the survival rate of glioma patients (AUC = 0.845) (Fig. 4H). We also conducted KM (*P* < .001) (Fig. 4E) and ROC curve (AUC = 0.805) (Fig. 4I) analyses on the samples from CGGA database to verify the accuracy of the results. Next, we used the RSs to draw a heat map. The results showed that in the TCGA dataset, 7 lncRNAs, namely, AC083799.1, AC062021.1, SNHG6, LINC00507, FAM66C, DGCR10, and LINC00641, were highly expressed in high-risk and low-risk patients (Fig. 4J). Both groups were closely related to the WHO grade (*P* < .001), age (*P* < .001), IDH status (*P* < .001), and 1p/19q codel status (*P* < .001). These analyses showed that the genes we selected and the prognostic RS models had a good prognostic value.

### 3.5. RS is closely related to clinicopathology

**Figure 5.**
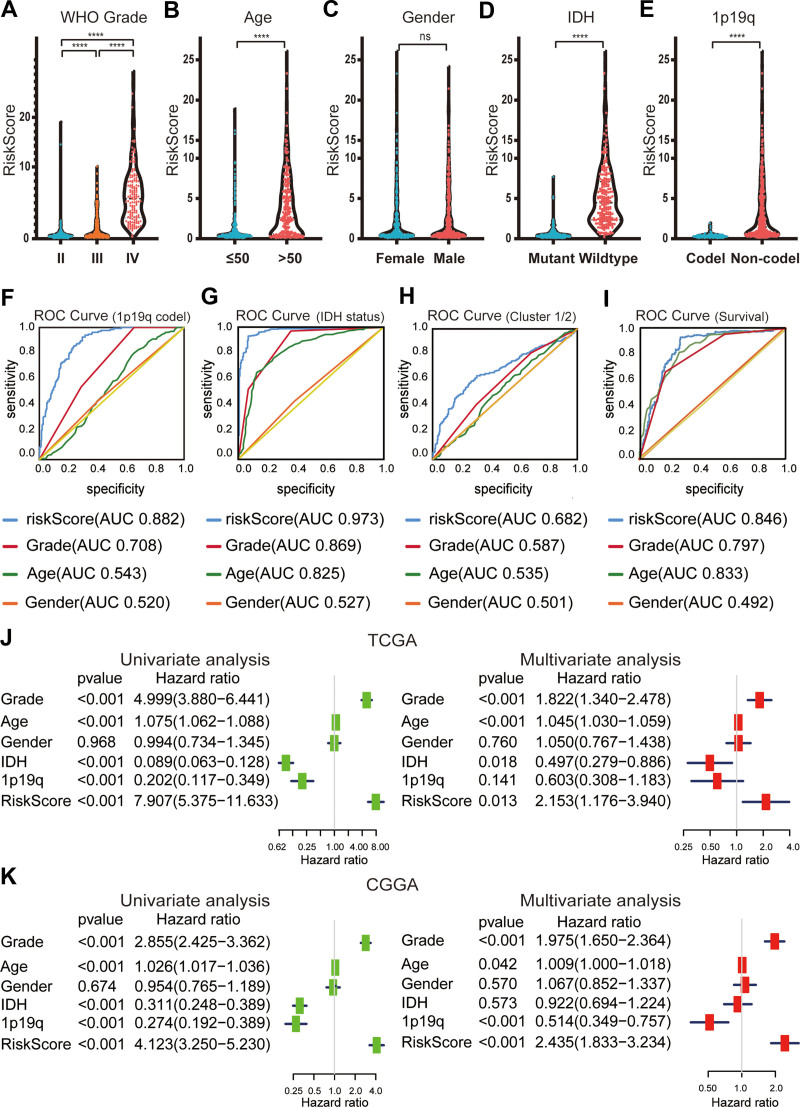
Comparison of risk scores and clinicopathological features between differences in OS of different grades of glioma. (A–E) Distribution of risk scores in the dataset from TCGA stratified by WHO grade (A), Age (B), sex (C), IDH status (D), and 1p/19q codel status (E). ns no significance, *P < .05 and ****P < .0001. (F–I) The predictive efficiency of the risk signature, WHO grade, and age on the 1p/19q codel status (F), IDH-mutant status (G), G1/2 subgroups (H), and survival rate (I) showed by ROC curves. (J and K) Univariate and multivariate Cox regression analyses of the association between clinicopathological factors (including the risk score) and overall survival of patients in TCGA (J) and CGGA (K). CGGA = Chinese glioma genome atlas, OS = overall survival, ROC = receiver operating characteristic, TCGA = the cancer genome atlas.

The RS model was used to test for each clinicopathological feature. The WHO grade (*P* < .0001), 1p/19q codel status (*P* < .0001), age (*P* < .0001), and IDH status (*P* < .0001) were significantly different between the 2 groups (Fig. 5A–E). The ROC curve showed that RS could accurately predict the 1p/19q codel status (AUC = 0.882), IDH mutation status (AUC = 0.973), G1/2 subgroup (AUC = 0.682), and survival rate of glioma patients (AUC = 0.846). It is worth noting that RS had a higher prediction accuracy than age and WHO grade (Fig. 5F–I).

Univariate and multivariate Cox regression analyses of TCGA database showed that RS, age, and WHO grade were related to OS (Fig. 5J). The verification dataset (CGGA dataset) yielded similar results (Fig. 5K). These findings suggest that the RS of lncRNAs related to abnormal glycosylation is an independent prognostic predictor in glioma patients.

### 3.6. Analysis of the expression of glycation-related lncRNAs

**Figure 6.**
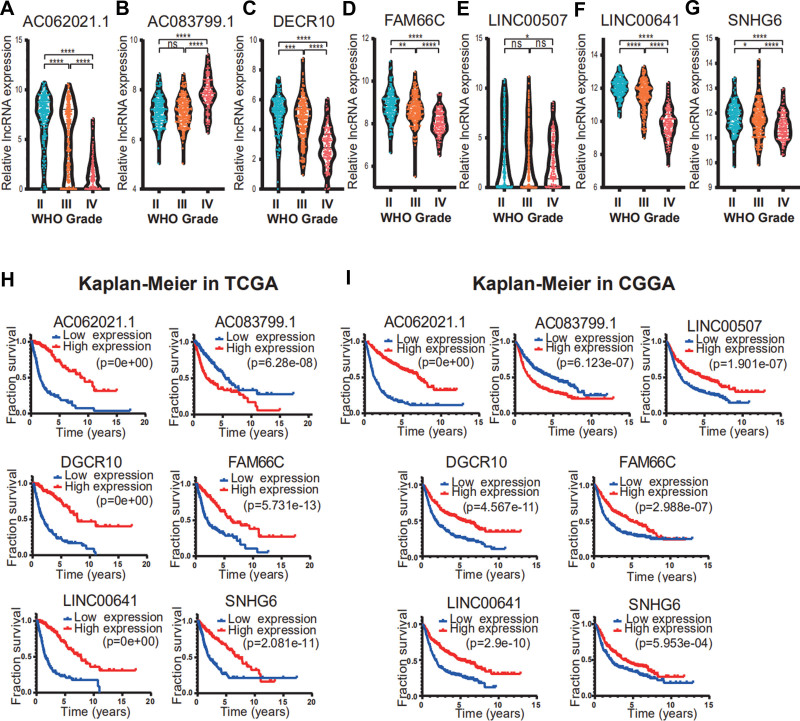
The correlation of 7 glycosylated lncRNAs with the development and prognosis of glioma. (A–G) The expression levels of 7 glycosylated lncRNAs in gliomas with different WHO grades. (H and I) Kaplan–Meier survival analysis of 7 glycosylation-related lncRNAs in TCGA (H) and CGGA (I). CGGA = Chinese glioma genome atlas, lncRNAs = long noncoding RNA, TCGA = the cancer genome atlas.

To evaluate the expression levels of the 7 lncRNAs related to abnormal glycosylation in TCGA dataset, we visualized their expression trends. According to the WHO classification for gliomas, only 1 lncRNA (LINC00507) was found to have no difference at different levels, whereas the other lncRNAs were significantly different (Fig. 6A–G).

### 3.7. Analysis and verification of prognosis and survival predictions for the glycation-related lncRNAs

Next, we examined the prognostic potential of the 7 lncRNAs in the risk model by KM analysis and found that lncRNAs could predict prognosis to varying degrees. The survival time of patients with low AC083799.1 expression in TCGA database was significantly higher than that of patients with high expression. The survival time of patients in the high and low LINC00507 expression groups were not significantly different. The survival time of the remaining patients in the low expression groups were significantly lower than that of patients in the high expression groups (Fig. 6H). The results obtained from the CGGA database were similar to those obtained from the TCGA database. However, the survival time of patients in the high and low LINC00507 expression groups were also significantly different (Fig. 6I).

### 3.8. Verification of the expression trend of glycosylation-related lncRNAs

**Figure 7.**
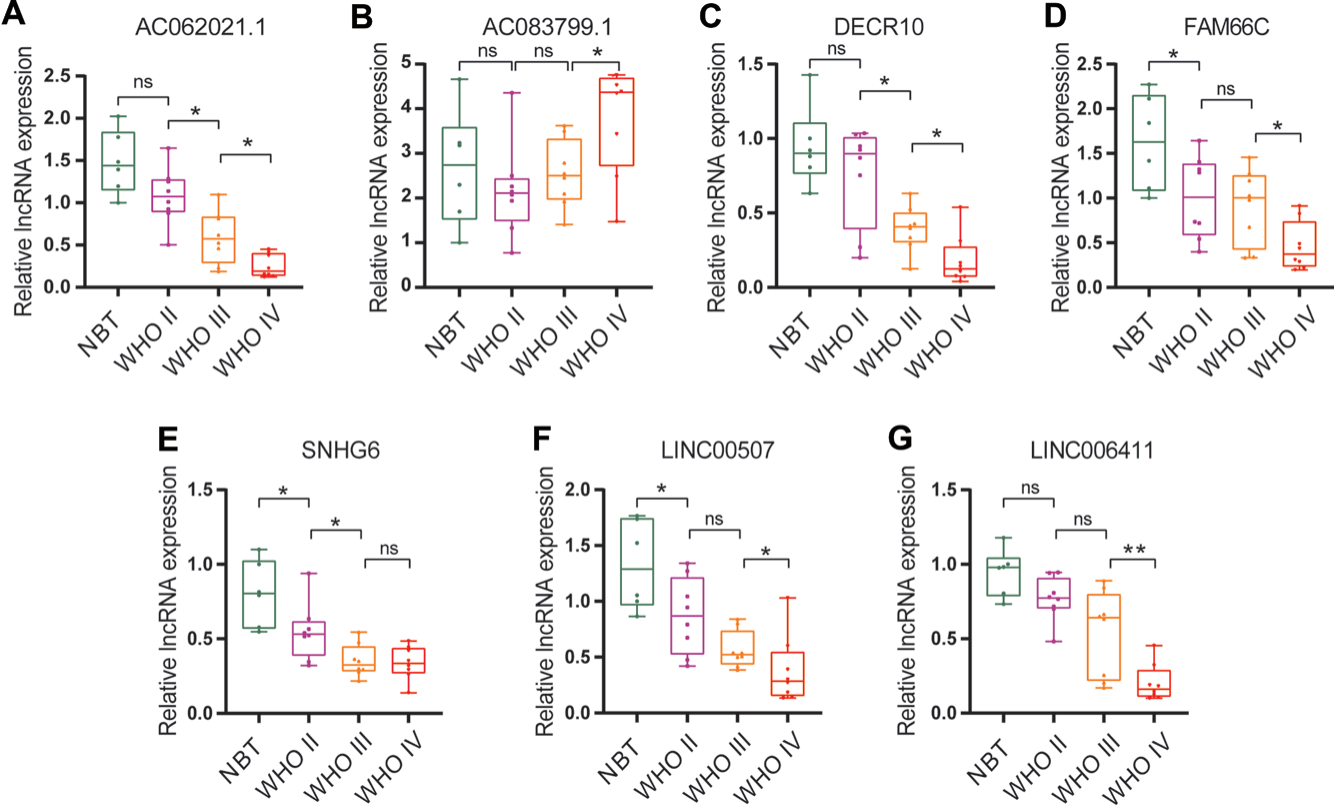
RT-qPCR assay results of 7 glycosylated lncRNAs in glioma tissues of different pathological grades (A–G). lncRNAs = long noncoding RNA.

Ultimately, 24 glioma tissue samples and 6 brain tissue samples were examined by qRT-PCR. The expression levels of AC062021.1, SNHG6, LINC00507, FAM66C, DGCR10, and LINC00641 were significantly lower in glioma tissues than in normal brain tissues (*P* < .05) and gradually increased with the degree of malignancy. In contrast, the expression level of AC083799.1 was significantly higher in glioma tissues than in normal brain tissue (*P* < .05) and increased with the degree of malignancy (Fig. 7A–G). These results verified the accuracy of our research.

## 4. Discussion

Glioma is the most common brain tumor.^[1,29,30]^ GBM is highly invasive and associated with high deterioration and a poor prognosis.^[1,31]^ Currently, the main treatment method is surgery combined with radiotherapy and chemotherapy, but the effect is not ideal.^[32,33]^ Research has shown that the survival rate has not improved significantly in recent years.^[34]^ Therefore, it is of great significance to explore potential therapeutic targets and prognostic indicators of gliomas.

LncRNAs were focused by an increasing number of researchers, which have been proven to exert multiple signal transduction and control functions that affect glioma mutation, occurrence and development, especially immunity^[35]^ and inflammation.^[36]^ In recent years, methylation-related lncRNAs have been shown to be useful in the early diagnosis of gliomas and prediction of glioma outcomes. For example, lncRNA GAS5 inhibits multiple malignant phenotypes of glioma by upregulating PRC2 and promoting methylation targeting miR-424.^[37]^ Six lncRNAs, including AC005013.5 and UBE2R2-AS1, have been shown to be involved in immune-related biological processes and pathways related to the occurrence of glioblastoma.^[38]^ In addition, recent studies have found that protein glycosylation plays an important role in glioma progression.^[39]^ Protein glycosylation can affect the stability, location and secretion of proteins and in turn plays a key role in cellular functions.^[40,41]^ Many glioma-related signals and effector proteins must be glycosylated. lncRNAs related to glycosylation have also been reported to be involved in glioma development. For example, upregulation and knockdown of SNHG14 in glioma cells inhibits glycolysis and proliferation.^[42]^

In this study, 2 glioma subgroups, G1/2, were identified by consistent clustering based on lncRNAs related to abnormal glycation. The G1 subgroup was associated with disease progression, pathological characteristics and with patient prognosis. In addition, we found that 20 abnormal glycosylation-related lncRNAs were associated with gliomas. We retained 7 genes from the LASSO regression analysis and used them to derive a prognostic RS, of which LINC00507 and AC083799.1 were.

upregulated with a positive regression coefficient, and SNHG6, FAM66C, LINC00641, DGCR10, and AC062021.1 were downregulated with a negative regression coefficient. The AUCs of the RS ROC curve in the TCGA and CGGA datasets were 0.845 and 0.805, respectively, which were higher than the prediction accuracy of age and WHO grade. To further explore the function of 7 glycosylation-related lncRNAs, we visualized the expression trends and performed KM analysis based on TCGA and CGGA datasets. The expression differences of these 7 lncRNAs in different WHO grades were significant. The survival time of patients with low AC083799.1 expression was significantly longer than that of patients with high expression. The survival times of the low SNHG6, FAM66C, LINC00641, DGCR10, and AC062021.1 expression groups were significantly shorter than those of the high expression groups. Moreover, we performed RT-PCR to examine 24 glioma tissues.

LINC00507, AC083799.1, SNHG6, FAM66C, LINC00641, DGCR10, and AC062021.1 from different WHO grades and 6 normal brain tissues. These results were similar to those obtained from the TCGA database. Therefore, lncRNAs with abnormal glycosylation are promising targets for molecular targeted therapy.

These lncRNAs should be further explored in future studies regarding the clinical target therapy of glioma, due to the fact that molecular targeted therapy of lncRNA is still under development and only a few lncRNAs have been applied in clinical studies. There are several limitations in this research. For example, mass spectrometry may be more suitable for analyzing glycosylation patterns, and further exploration of the biological characteristics of lncRNA is needed.

Our research revealed the exact mechanism of glycosylation regulation in GBM and the genes involved. These results will enable the design of targeted drugs for the treatment of gliomas.

## 5. Conclusion

In conclusion, we successfully constructed a formula using lncRNAs related to abnormal glycosylation with powerful predictive functions and revealed the expression, function, and prognostic potential of these lncRNAs in gliomas. These lncRNAs may serve as novel biomarkers and therapeutic targets for glioma development. Our findings are important for further exploration of the role of glycosylation in glioma.

## Acknowledgements

The authors gratefully acknowledge the contributions of the CGGA and TCGA networks.

## Author contributions

**Data curation:** Haitao Luo.

**Funding acquisition:** Feng Liu.

**Investigation:** Xiang Wu.

**Project administration:** Xiang Wu.

**Software:** Haiyan Wang, Shiqi Li.

**Writing – original draft:** Xiang Wu.

**Writing – review & editing:** Xiang Wu, Shiqi Li.

## Supplementary Material












